# Neuroendocrine Breast Carcinoma: Interesting Images of an Underdiagnosed Entity

**DOI:** 10.3390/diagnostics14111133

**Published:** 2024-05-29

**Authors:** Christoforos Kosmidis, Kassiani Boulogeorgou, Panagiota Roulia, Marios Dagher, Georgios Anthimidis, Georgios Petrakis, Charilaos Koulouris, Stylianos Mantalovas, Styliani Laskou, Vasiliki Magra, Vasileios Alexandros Karakousis, Christina Sevva, Eleni Paschou, Vasileios Stergios, Stylianos Kosmidis, Chrysi Maria Mystakidou, Vasiliki Theodorou, Nikolaos Iason Katsios, Triantafyllia Koletsa, Konstantinos Sapalidis, Isaak Kesisoglou

**Affiliations:** 1 3rd Surgical Department, University General Hospital of Thessaloniki “AHEPA”, Aristotle University of Thessaloniki, 1st St. Kiriakidi Street, 54621 Thessaloniki, Greece; dr.ckosmidis@gamil.com (C.K.); mariosdag@gmail.com (M.D.); charilaoskoulouris@gmail.com (C.K.); steliosmantalobas@yahoo.gr (S.M.); stelaskou@gmail.com (S.L.); valia.magra@gmail.com (V.M.); alexanderkarakousis@gmail.com (V.A.K.); christina.sevva@gmail.com (C.S.); paschoueleni@gmail.com (E.P.); vasilis_stergios@hotmail.com (V.S.); sapalidiskonstantinos@gmail.com (K.S.); ikesis@hotmail.com (I.K.); 2 Department of Pathology, School of Medicine, Aristotle University of Thessaloniki, 54621 Thessaloniki, Greece; siliaboulog@gmail.com (K.B.); georgiospetrakismd@gmail.com (G.P.); tkoletsa@gmail.com (T.K.); 3 European Interbalkan Medical Center, 10 Asklipiou Street, 55535 Pylaia, Greece; anthimid@gmail.com; 4 Department of Medicine, Medical University of Plovdiv, y.B.Apo15A, 4002 Plovdiv, Bulgaria; stylianoskosmidis830@gmail.com; 5 Faculty of Health Sciences, Medical School, Aristotle University of Thessaloniki, 54124 Thessaloniki, Greece; chryssa2000@gmail.com (C.M.M.); baswtheodorou@hotmail.com (V.T.); 6 Faculty of Health Sciences, Medical School, University of Ioannina, 45110 Ioannina, Greece; nickkatsios@hotmail.gr

**Keywords:** breast, cancer, neuroendocrine, synaptophysin, chromogranin

## Abstract

Breast cancer is the most common type of cancer of the female gender. A rare subtype of breast cancer is the invasive breast carcinoma (IBC) with neuroendocrine (NE) differentiation. Its incident is believed to be 0.1% to 5% of all breast cancers. We report a rare case of a 66-year old woman who presented with an isolated nodule of the left breast. The patient underwent modified radical mastectomy. Pathology revealed invasive breast carcinoma with neuroendocrine differentiation. Invasive breast carcinoma is an extremely rare group of neoplasms, the exact frequency of which cannot be determined with current data. Therefore, it is necessary for future studies to focus on the pathophysiology of this subtype of breast cancer and on the potential therapeutic approaches.

Breast cancer is the most common type of cancer in females, with an incidence of 24% as reported by WHO Classification of Tumors Editorial Board, 2019. Invasive breast carcinoma encompasses a wide variety of morphologic subtypes. It can present in both women and men, but it is far more common in women. A rare subtype of breast cancer is the invasive breast carcinoma (IBC) with neuroendocrine (NE) differentiation. The incidence of such tumors is not precisely determined, but it is believed to be less than 1% of all neuroendocrine neoplasms and 0.1% to 5% of all breast carcinomas [1]. Their clinical presentation is similar to the rest of breast carcinomas. Diagnosis is based on the presence of the histopathological characteristics of neuroendocrine differentiation in the invasive component and is confirmed when the NE-markers chromogranin and synaptophysin are expressed [2,3]. Due to its rarity and suspected underdiagnosis, there is no universally agreed upon treatment protocol. They are usually treated as a breast carcinoma NOS—specifically with mastectomy followed by radiotherapy and chemotherapy plus endocrine therapy if necessary. We present an exceptional case of invasive neuroendocrine breast carcinoma co-existent with background ductal carcinoma in situ.

IBC-NE usually presents as an isolated breast nodule. It can cause local symptomatology such as bloody areolar discharge, nipple retraction, and painful axillary adenopathy. Clinical signs caused by metastatic disease are rare. Ectopic hormonal secretion is even rarer. Clinical signs may be absent and the disease presents during routine mammography as a hyperdense, irregularly shaped solitary mass [1]. Breast ultrasound is the next step in diagnostic imaging, which reveals an irregular hypoechoic lesion. An MRI scan can also be performed to detect a hypointense irregular lesion on T1-weighted sequences. The risk factors for IBC-NE are the same as for the other types of breast cancer. The most important factors are menopausal status and family history [4]. A significant exposure to estrogens and early menarche or late menopause can also increase the risk [1].

We highlight the case of a 66-year-old woman who presented at the outpatient breast department with an isolated nodule of the left breast. The clinical examination revealed a palpable breast lump between 3 and 5 o’clock. There were palpable left axillary lymph nodes. The patient mentions menarche at the age of 14 years old and menopause at the age of 53 years old. She had 2 full-term pregnancies, the first one at the age of 27, no history of miscarriage, and she had no history of hormonal contraception use. Other past medical history included asthma and osteopenia. The patient had mammography and breast ultrasound. These revealed two masses in the upper outer quadrant of the left breast, approximately 3 cm in diameter, one of which was characterized as BIRADS 5 (Figure 1). Two enlarged axillary lymph nodes were also found in the ipsilateral breast. Complete laboratory tests were performed, including tumor markers, the values of which were within normal limits. It was decided to proceed with surgical excision. 

Little is known about the ideal therapeutic approach of IBC-NE. In most cases, the first step is surgical resection with partial or total mastectomy with or without axillary lymph node dissection [1,5]. Surgical excision may be followed by radiotherapy, chemotherapy, and endocrine therapy [6]. Most chemotherapy regiments include platinum agents and etoposide or taxanes, and an anthracycline plus taxane regimen is also commonly used. Endocrine therapy is usually required for 5–10 years and it usually involves aromatase inhibitors. Anti-HER2 therapy with trastuzumab is also used in HER2-positive patients [5]. Follow-up occurs every 3–4 months in the first 2 years and every 6–8 months from the third year onwards. Annual mammography, ultrasound, and MRI of the breast are recommended. In this case, the patient had sentinel lymph node marking with a radioactive isotope pre-operatively. Two masses of the left breast were resected and sent for intraoperative fresh frozen section (Figure 2a). Macroscopically, the mass was pale, semi-solid, nodular in configuration and measured 4 × 2, 5 × 2.3 cm. Frozen sections revealed invasive breast carcinoma. The sentinel lymph node was identified using a Geiger Muller counter, resected, and sent for fresh frozen section. These revealed metastatic foci of the carcinoma. The decision was made to proceed with modified radical mastectomy (Figure 2b). Two vacuum drains were placed, an axillary and pre-pectoral drain, respectively. The patient had an uneventful postoperative recovery. The drains were removed on the 2nd postoperative day and the patient was discharged on the 3rd postoperative day. Based on the biopsy findings, the patient was eligible to receive adjuvant chemotherapy and hormone therapy.

Both specimens were fixed in 10% formalin buffer and sections from the aforementioned mass as well as from a second stellate lesion that was at a distance of 1.7 cm from the first, sized 2.5 cm, were taken. Hematoxylin and eosin (H&E)-stained sections from the larger tumor confirmed the diagnosis of invasive breast carcinoma (IBC) occupying about 90% of the tumor surface with coexistent ductal carcinoma in situ in a few small areas at the periphery of the tumor. The invasive carcinoma was characterized by variably sized clusters and nests, of medium to large cuboidal cells with pale cytoplasm, a high nuclear/cytoplasmic ratio, fine nuclear chromatin, or inconspicuous nucleoli (Figure 3a). Mitotic rate was up to 11 mitoses/10 high power fields (with field diameter at magnification ×400 equal to 0.56 μm). Occasionally, tumor cells were polarized in forming rosette-like structures (Figure 3b). Tumor necrosis was evident in central areas of some neoplastic cell groups as well as within foci of carcinoma in situ. Perineural and lymphatic invasion were detected focally. Immunohistochemical study showed a neuroendocrine nature by expressing synaptophysin (Figure 3c) and to a considerable extent chromogranin (Figure 3d) by the tumor cells. NE markers were expressed by carcinoma in situ, as well. On the other hand, H&E sections from the second lesion showed hyperplastic fibrocystic changes. Sections from the radical mastectomy specimen revealed a small (0.5 cm), residual focus of invasive carcinoma and ductal carcinoma in situ of intermediate and high nuclear grade. Axillary lymphadenectomy revealed a total of 16 lymph nodes, measured from 0.1 cm to 2 cm in diameter, which were all free of metastases. In regard to predictive markers, strong estrogen receptor (ER) (Figure 3e) and progesterone receptor (PR) positivity was detected in >95% and 90% of the neoplastic cells, respectively, whereas immune stain for HER2 protein was negative (HER2 0). The intensity of ER stains was high H 3+ in Allred score and the intensity of PR stains was moderate to high, 2–3 in Allred score. The Ki67 proliferative index was heterogeneous, ranging from 20% to 60% (Figure 3f). In summary, the histological and immunohistochemical findings were consistent with invasive breast carcinoma (IBC/NST) with neuroendocrine (NE) differentiation along with ductal carcinoma in situ, in extent of 25% of the total tumor surface, of intermediate and high nuclear grade. We present this case not only for its rarity but also to highlight the different terms used in the English literature for breast carcinoma that exhibit expression of NE markers with inconsistent nomenclature, namely invasive breast carcinoma with NE features, invasive breast carcinoma with NE differentiation, and NEBC [4,7]. The latest WHO classification of breast tumors mentions three types of neuroendocrine breast tumors: 1. neuroendocrine tumor, grade 1 (NET, G1) 2. neuroendocrine tumor, grade 2 (NET, G2), and 3. neuroendocrine breast carcinoma (NEBC), in line with the classification of neuroendocrine tumors of other anatomical sites [8,9]. Invasive breast carcinomas with neuroendocrine differentiation are a rare heterogeneous group of neoplasms. NECB is a type of breast carcinoma characterized by the presence of NE histological features and the positivity to NE markers, such as chromogranin and synaptophysin [10]. NE markers can be expressed to some extent in many IBCs without being a criterion to set the diagnosis of neuroendocrine carcinoma. The previous used cut-off of >50% is considered arbitrary. Therefore, the diagnosis of NEBC is based on the combined morphological and immunohistochemical features suggestive of neuroendocrine nature. However, there is still confusion in the literature about the threshold for distinguishing an invasive breast carcinoma with neuroendocrine differentiation (IBC-NE) from an NEBC. Its incidence is estimated to be 0.1% to 5% of all breast carcinomas but they are often underdiagnosed [1]. This type of cancer is extremely rare. Because of these aforementioned factors, the knowledge we have about IBC-NEs is limited. It is necessary for more research to be conducted, especially regarding their pathology and optimal treatment. The results may contribute to a new and more effective therapeutic approach.

## Figures and Tables

**Figure 1:**
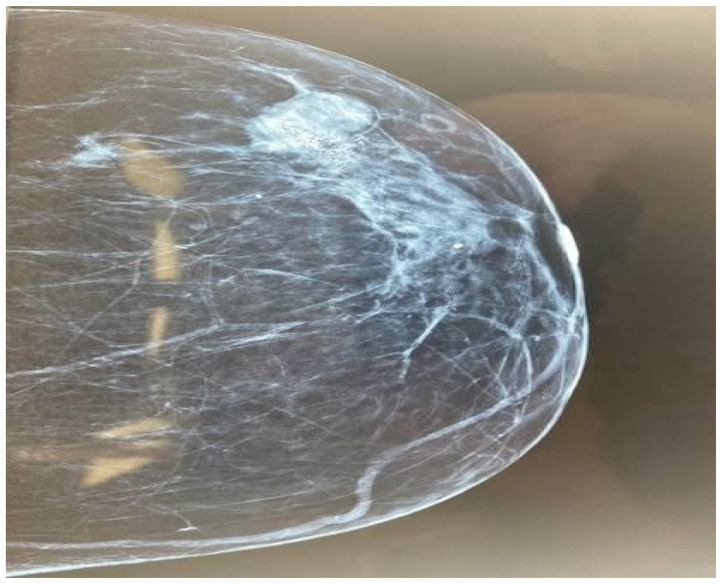
Mammography imaging of the left breast.

**Figure 2:**
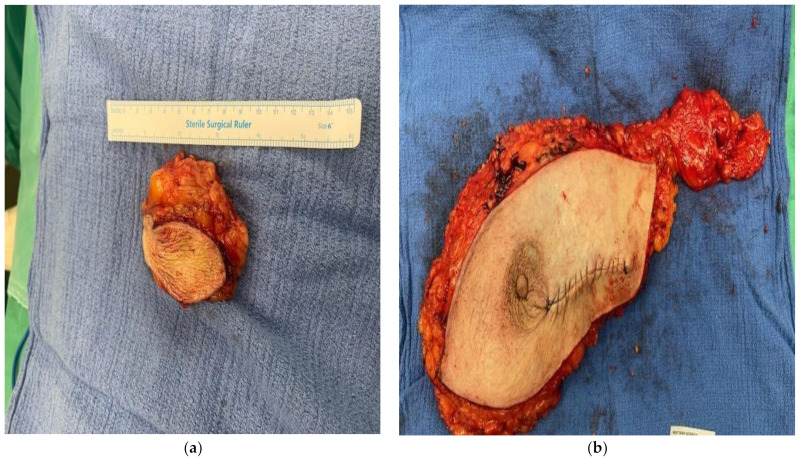
(**a**) Mass of the left breast sent for intraoperative fresh frozen section. (**b**) Left breast and lymph nodes of the left axilla.

**Figure 3:**
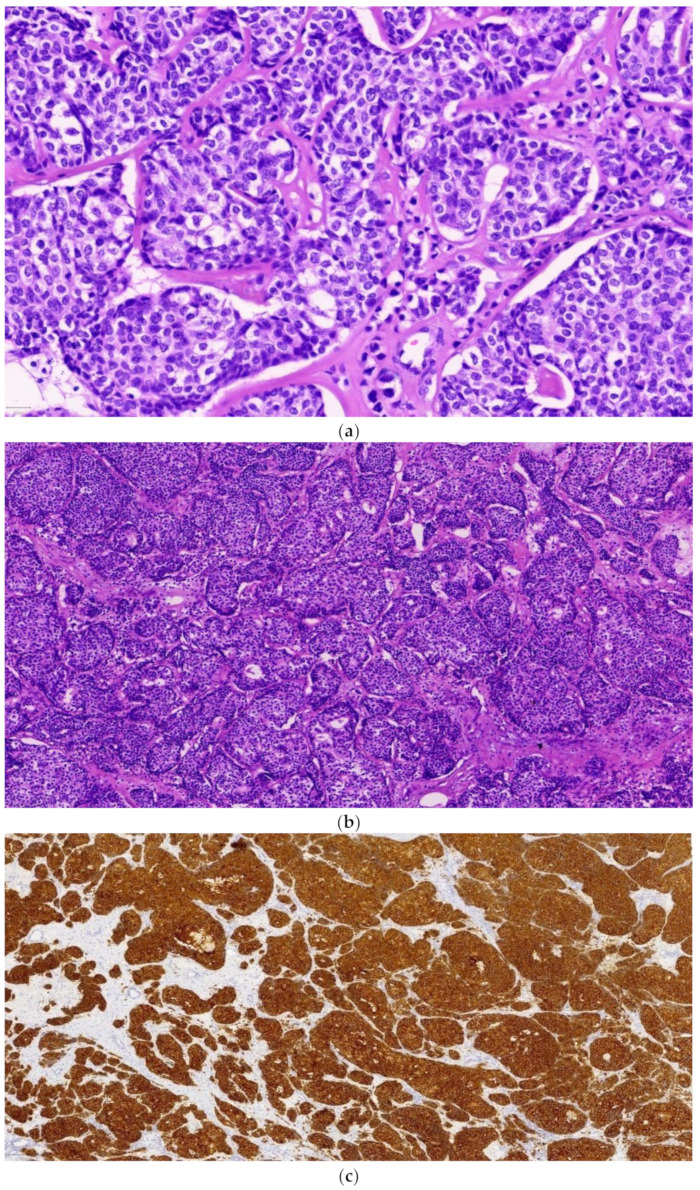

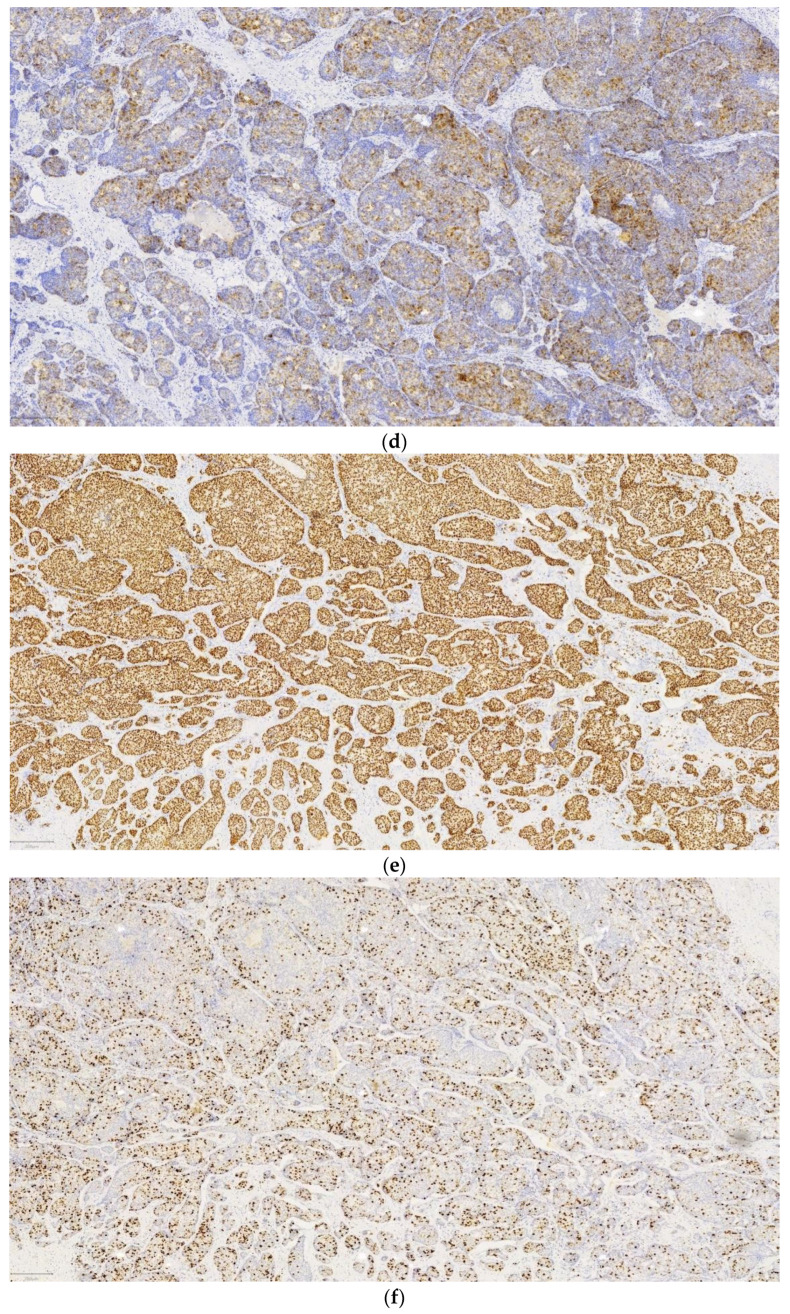
(**a**) Breast carcinoma presenting neuroendocrine morphology. (**b**) Breast carcinoma presenting neuroendocrine morphology. (**c**) Immunohistochemical expression of synaptophysin. (**d**) Immunohistochemical expression of chromogranin. (**e**) Immunohistochemical expression of estrogen receptors. (**f**) Immunohistochemical expression of a high Ki67 proliferative index.

## Data Availability

All data used in this case report is available from the corresponding author and can be provided upon reasonable request.
